# Bioinformatics analysis of potential common pathogenic mechanisms for COVID-19 infection and primary Sjogren’s syndrome

**DOI:** 10.3389/fimmu.2022.938837

**Published:** 2022-07-26

**Authors:** Hong Luo, Xia Zhou

**Affiliations:** ^1^ Department of Tuberculosis and Respiratory, Wuhan Jinyintan Hospital, Tongji Medical College, Huazhong University of Science and Technology, Wuhan, China; ^2^ Hubei Clinical Research Center for Infectious Diseases, Wuhan, China; ^3^ Wuhan Research Center for Communicable Disease Diagnosis and Treatment, Chinese Academy of Medical Sciences, Wuhan, China; ^4^ Joint Laboratory of Infectious Diseases and Health, Wuhan Institute of Virology and Wuhan Jinyintan Hospital, Chinese Academy of Sciences, Wuhan, China

**Keywords:** primary Sjogren’s syndrome, COVID-19, differentially expressed genes, hub genes, pathogenesis

## Abstract

**Background:**

Accumulating evidence has revealed that the prevalence of Coronavirus 2019 (COVID-19) was significantly higher in patients with primary Sjogren’s syndrome (pSS) compared to the general population. However, the mechanism remains incompletely elucidated. This study aimed to further investigate the molecular mechanisms underlying the development of this complication.

**Methods:**

The gene expression profiles of COVID-19 (GSE157103) and pSS (GSE40611) were downloaded from the Gene Expression Omnibus (GEO) database. After identifying the common differentially expressed genes (DEGs) for pSS and COVID-19, functional annotation, protein-protein interaction (PPI) network, module construction and hub gene identification were performed. Finally, we constructed transcription factor (TF)-gene regulatory network and TF-miRNA regulatory network for hub genes.

**Results:**

A total of 40 common DEGs were selected for subsequent analyses. Functional analyses showed that cellular components and metabolic pathways collectively participated in the development and progression of pSS and COVID-19. Finally, 12 significant hub genes were identified using the cytoHubba plugin, including CMPK2, TYMS, RRM2, HERC5, IFI44L, IFI44, IFIT2, IFIT1, IFIT3, MX1, CDCA2 and TOP2A, which had preferable values as diagnostic markers for COVID-19 and pSS.

**Conclusions:**

Our study reveals common pathogenesis of pSS and COVID-19. These common pathways and pivotal genes may provide new ideas for further mechanistic studies.

## Introduction

Primary Sjogren’s syndrome (pSS) is one of the most common systemic autoimmune disorders, frequently accompanied by a variety of specific autoantibodies, such as antinuclear antibodies (ANAs), antibodies against Ro/Sjogren’s syndrome-related antigen A (SSA) and La/Sjogren’s syndrome-related antigen B(SSB), and hypergammaglobulinemia (1, 2). The prevalence of pSS is about 60 cases per 100,000 inhabitants (3) and there is a significant gender distribution difference, with the number of male patients to female patients ratio being about 1:9 (4). It is marked by lymphocytic infiltration of exocrine glands, such as lacrimal glands, salivary glands and other exocrine glands, characterized by oral and ocular dryness. At least one-third of patients with pSS may have multiple organ function impairment, such as severe thrombocytopenic purpura, primary biliary cirrhosis, and interstitial pneumonia, which can seriously compromise the patient’s prognosis, and 5% of patients may develop lymphoma (5).

The etiology and pathogenesis of pSS are still not fully elucidated and may be related to various factors such as infection, genetics and sex hormone abnormalities. Among them, viral infections are more closely related to pSS. Epstein-Barr virus (EBV), cytomegalovirus (CMV) and hepatitis C virus (HCV) may play an important role in the pathogenesis of pSS.

EBV, a DNA virus, was the first virus identified in association with pSS. EBV can affect the host immune system by directly infecting lymphocytes and indirectly regulating the expression of viral antigens through immunomodulatory mechanisms (6). The DNA component of EBV has been found to be detectable in the epithelial cells of saliva and lacrimal glands of patients with pSS (7). Studies have reported that EBV can induce autoimmune disorders in pSS through type I interferon, molecular mimicry and ectopic lymphoid-like structures (ELS),a feature of pSS pathogenesis. EBV promotes the development and progression of pSS by inducing TLR to promote IFN-I production by dendritic cells (8, 9). In addition, the molecular mimicry between pSS autoantigens and EBV-associated antigens in the serum of pSS patients suggests that EBV infection may be involved in pathogenesis through molecular mimicry mechanism (10). Moreover, EBV can invade the ELS and thus contribute to the growth and differentiation of self-reactive B cells in pSS patients (11). CMV is a double-stranded DNA virus. It was found that CMV IgG concentrations were higher in the control group than in the pSS patient group, implying that CMV infection may be associated with the development of pSS, however, this needs to be confirmed by further clinical studies (12). HCV is a RNA virus capable of causing chronic hepatitis, cirrhosis and hepatocellular carcinoma. The pSS-characteristic salivary gland lymphocyte infiltration was found in patients with hepatitis C (13). Therefore, it was hypothesized that HCV infection might be associated with the development of SS and further studies are needed to confirm the relationship between HCV and pSS. However, although viruses such as EBV, CMV and HCV have been found to influence the pathogenesis of pSS, there are still relatively few studies focusing on COVID-19 and pSS.

Currently, Coronavirus 2019 (COVID-19), caused by severe acute respiratory syndrome coronavirus 2 (SARS-CoV-2), is ravaging the world, which is posing an ongoing challenge to global health (14). Globally, as of 8 April 2022, there have been 494,587,638 confirmed cases of COVID-19, including 6,170,283 deaths (https://covid19.who.int/). In addition to imposing a severe burden on global healthcare systems, the epidemic is also posing a serious challenge to the management of patients with “inflammatory autoimmune systemic diseases”, including primary Sjogren’s syndrome (15, 16). Multiple studies have reported that a significantly higher prevalence of COVID-19 has been observed in patients with pSS than in the general population (17, 18). Immune dysfunction, as well as the use of immunosuppressive therapies, have been reported to predispose pSS patients to severe bacterial and viral infections (18), however, the underlying mechanism of this phenomenon is still not fully elucidated.

Exploring the common transcriptional profile of pSS and COVID-19 may provide new insights into common pathogenesis of the two diseases. In this study, we aimed to identify pivotal genes associated with the pathogenesis of pSS complicated with COVID-19. Two datasets downloaded from the GEO database (GSE30999 and GSE28829) were analyzed. Integrated bioinformatics and enrichment analysis were used to identify common DEGs and their functions in COVID-19 and pSS. In addition, a PPI network was constructed using the STRING database and Cytoscape software (version 3.9.1) to analyze the gene modules and identify hub genes. Finally, we identified 12 important hub genes and further constructed TF-gene regulatory network and TF-miRNA regulatory network for these genes. The hub genes identified in this study between COVID-19 and pSS are expected to provide new insights into the biological mechanisms of these two diseases.

## Materials and methods

### Datasets preparation

GEO (www.ncbi.nlm.nih.gov/geo) is a large database containing gene expression for multiple diseases, which is publicly available and free of charge (19). GSE157103 (20) dataset contains 100 COVID-19 samples and 26 non-COVID-19 samples, which used high throughput sequencing technology based on Illumina NovaSeq 6000 platform. GSE40611 (21) dataset consists of 17 pSS tissues and 18 control tissues, which was based on Affymetrix Human Genome U133 Plus 2.0 Array platform.

### Identification of shared DEGs between COVID-19 and primary Sjogren’s syndrome

GEO2R (22) (www.ncbi.nlm.nih.gov/geo/geo2r/) is an online web-based tool that can be employed to compare and analyze gene expression between different sample groups. Networkanalyst (23) (www.networkanalyst.ca/) is an online visual analytics platform that enables gene expression differential analysis and enrichment analysis, meta-analysis, protein-protein interaction network analysis, and integrated analysis of multiple datasets. In this study, Networkanalyst was used to identify DEGs for GSE157103 and we employed GEO2R to analyze DEGs for GSE40611. Genes with adjusted P-value < 0.05 and |log_2_ fold change (log_2_FC)| > 1.0 were considered DEGs. The R language package VennDiagram (24) was used to obtain shared DEGs between the GSE157103 and GSE40611 datasets.

### Gene ontology and KEGG enrichment analysis

KEGG Orthology Based Annotation System (25) (KOBAS; http://bioinfo.org/kobas) is a database developed by Peking University for annotation and identification of enriched pathways and diseases. Gene ontology and KEGG enrichment analysis were performed to analyze the potential function of DEGs by using the KOBAS 3.0 database. Adjusted P-value < 0.05 was considered statistically significant.

### Construction of protein–protein interaction network and module analysis

Search Tool for the Retrieval of Interacting Genes (STRING (26); http://string-db.org) (version 11.5) is a database for the study of protein interactions with information on more than 14,000 species, more than 60 million proteins, and more than 20 billion interactions, which include both direct physical interactions as well as indirect functional correlations. The protein-protein interaction (PPI) interaction network of the common DEGs was constructed by STRING with an interaction score > 0.4. Cytoscape software (27) (version 3.9.0) was used to visualize the PPI network and we used the Cytoscape plug-in Molecular Complex Detection (MCODE (28)) to analyze core functional modules. The parameters were set as follows: degree cutoff = 2, max depth = 100, node score cutoff = 0.2 and K-core = 2. Then KOBAS 3.0 was applied to carry out KEGG and GO analysis of the genes in each module.

### Identification and analysis of hub genes

The hub genes were selected by a plug-in cytoHubba (29) of Cytoscape and then seven algorithms (Closeness, MCC, Degree, MNC, Radiality, Stress and EPC) were used to confirm the final hub genes, which were visualized by Venn diagram. GeneMANIA (30) (http://genemania.org), an online tool that can predict gene interactions, was utilized to construct a co-expression network of identified hub genes.

### Construction of TF-gene regulatory network and TF-miRNA regulatory network

In this study, TF-gene regulatory network and TF-miRNA regulatory network were constructed by utilizing the Networkanalyst platform. In the case of TF-gene regulatory network, the ENCODE (31) (https://www.encodeproject.org/) database, which is included in the Networkanalyst platform, was been used. As for TF-miRNA regulatory network, it was acquired from the RegNetwork (32) (http://www.regnetworkweb.org) database, which is incorporated in the NetworkAnalyst platform.

### ROC curves of hub genes

ROC curves were constructed and the area under the ROC curve (AUC) was calculated separately to evaluate the diagnostic performance of the hub genes on COVID-19 and pSS using the R packages “pROC” (33).

## Results

### Identification of DEGs and shared genes between COVID-19 and primary Sjogren’s syndrome

**Figure 1:**
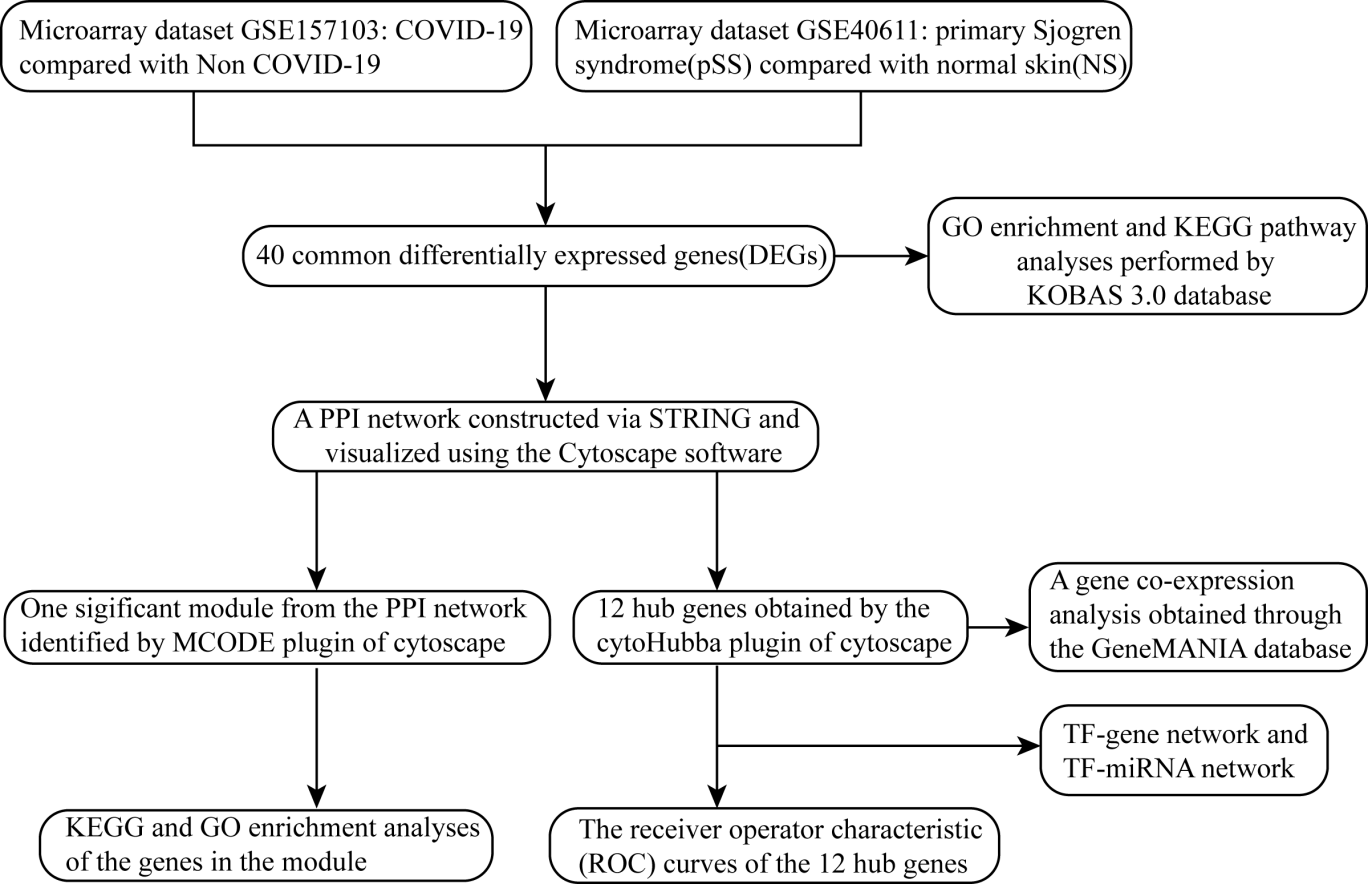
Workflow diagram of this study.

**Figure 2:**
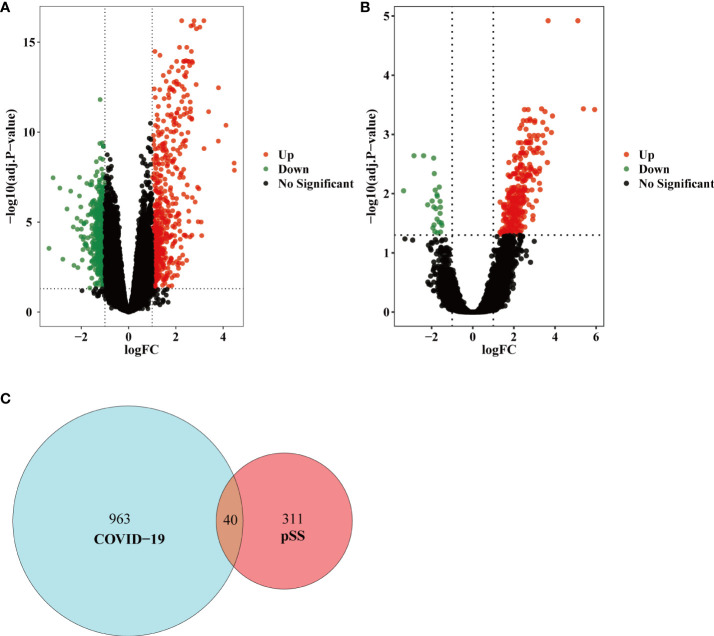
Volcano diagram and Venn diagram. **(A)** The volcano map of GSE157103. **(B)** The volcano map of GSE40611. Upregulated genes are colored in red; downregulated genes are colored in green. **(C)** The two datasets showed an overlap of 40 DEGs.

The overall flow chart of this study was shown in **Figure 1**. For GSE157103 dataset, a total of 1003 DEGs were identified, among which 554 genes were up-regulated and 449 genes were down-regulated (**Figure 2A**). Based on the GSE40611 dataset, we identified 351 DEGs including 291 upregulated genes and 60 downregulated genes (**Figure 2B**). Then by taking the intersection of DEGs of GSE157103 dataset and GSE40611 dataset, there were 40 shared DEGs selected, which were visualized by Venn diagrams (**Figure 2C**).

### GO and KEGG pathway enrichment analysis

**Figure 3:**
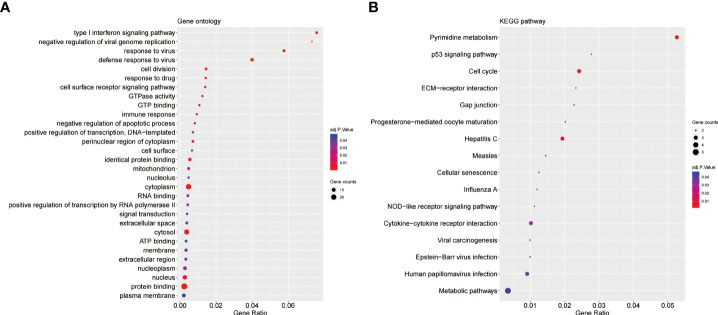
GO and KEGG enrichment analysis of common DEGs. **(A)**The enrichment analysis results of GO. **(B)**The enrichment analysis results of KEGG Pathway. Adjusted P-value < 0.05 was considered significant.

For GO enrichment analysis, the top five significant terms showed that the shared DEGs were mainly involved in protein binding cytoplasm, cytosol, nucleus and nucleoplasm (**Figure 3A**). In terms of KEGG pathway enrichment analysis, the top five significant terms were metabolic pathways, pyrimidine metabolism, cell cycle, hepatitis C and cytokine-cytokine receptor interaction. These results forcefully indicated that cellular component and metabolic pathways collectively participated in the development and progression of both inflammatory diseases (**Figure 3B**).

### Protein–protein interaction network analysis and submodule analysis

**Figure 4:**
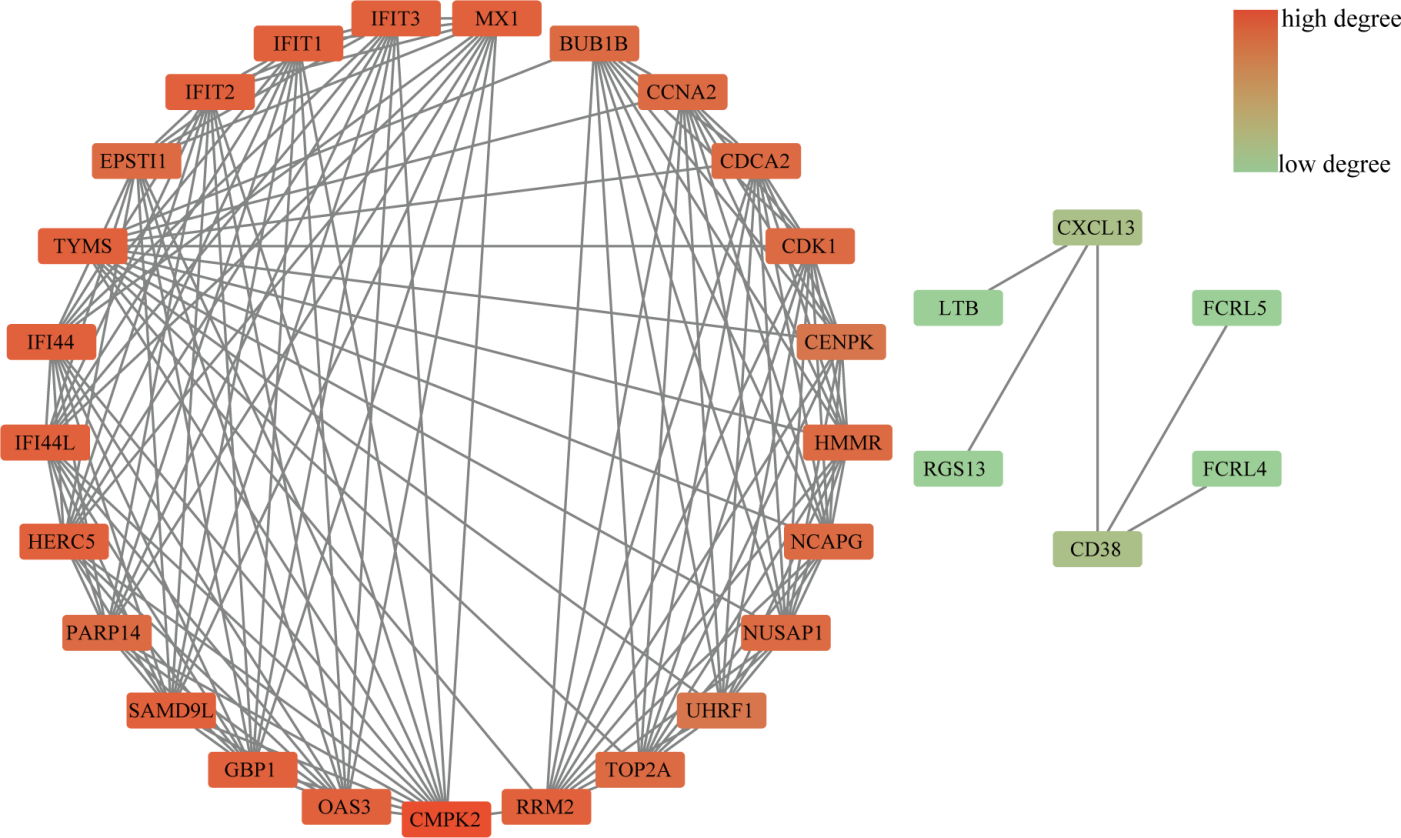
PPI network diagram. The redder the color of the gene in the network, the higher the connectivity of the gene with other genes.

**Figure 5:**
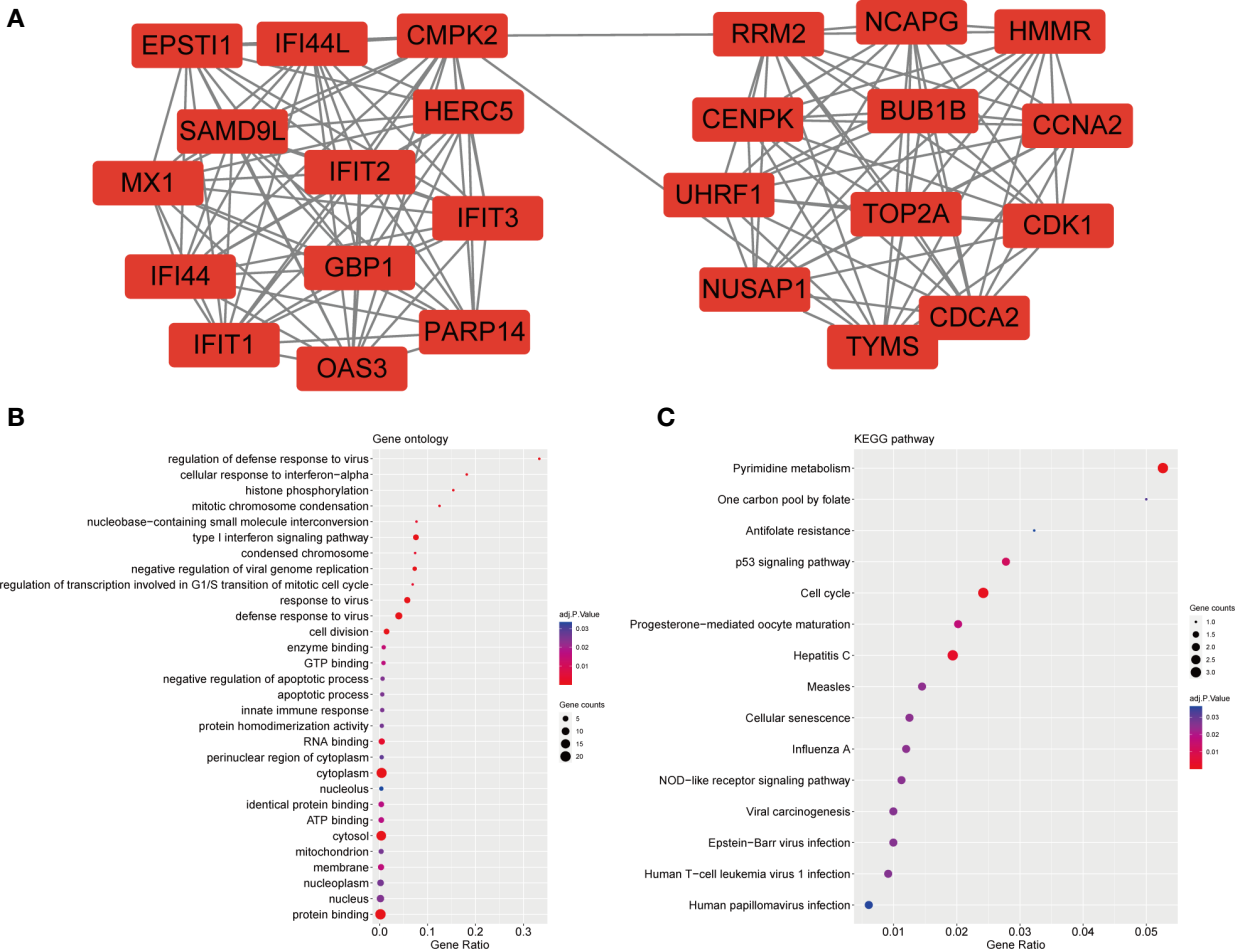
Significant gene module and enrichment analysis of the modular genes **(A)** A significant gene clustering module. **(B)** GO enrichment analysis of the modular genes. **(C)** KEGG enrichment analysis of the modular genes.

The PPI network included 40 nodes and 149 edges, of which the PPI enrichment P-value was lower than 1.0e − 16 (**Figure 4**). By visualizing the PPI network using Cytoscape software, the redder the color of the gene in the network, the higher the connectivity of the gene with other genes. A key gene module, including 25 shared DEGs, was obtained by applying the MCODE plug-in of Cytoscape (**Figure 5A**). GO enrichment analysis of these genes in the module showed that these genes were mainly associated with cytoplasm, defense response to virus, response to virus, cytosol and type I interferon signaling pathway (**Figure 5B**). KEGG enrichment analysis of these genes in the module indicated that these genes were mainly related to pyrimidine metabolism, cell cycle, p53 signaling pathway, progesterone-mediated oocyte maturation and cellular senescence (**Figure 5C**).

### Identification and functional analysis of hub genes

**Figure 6:**
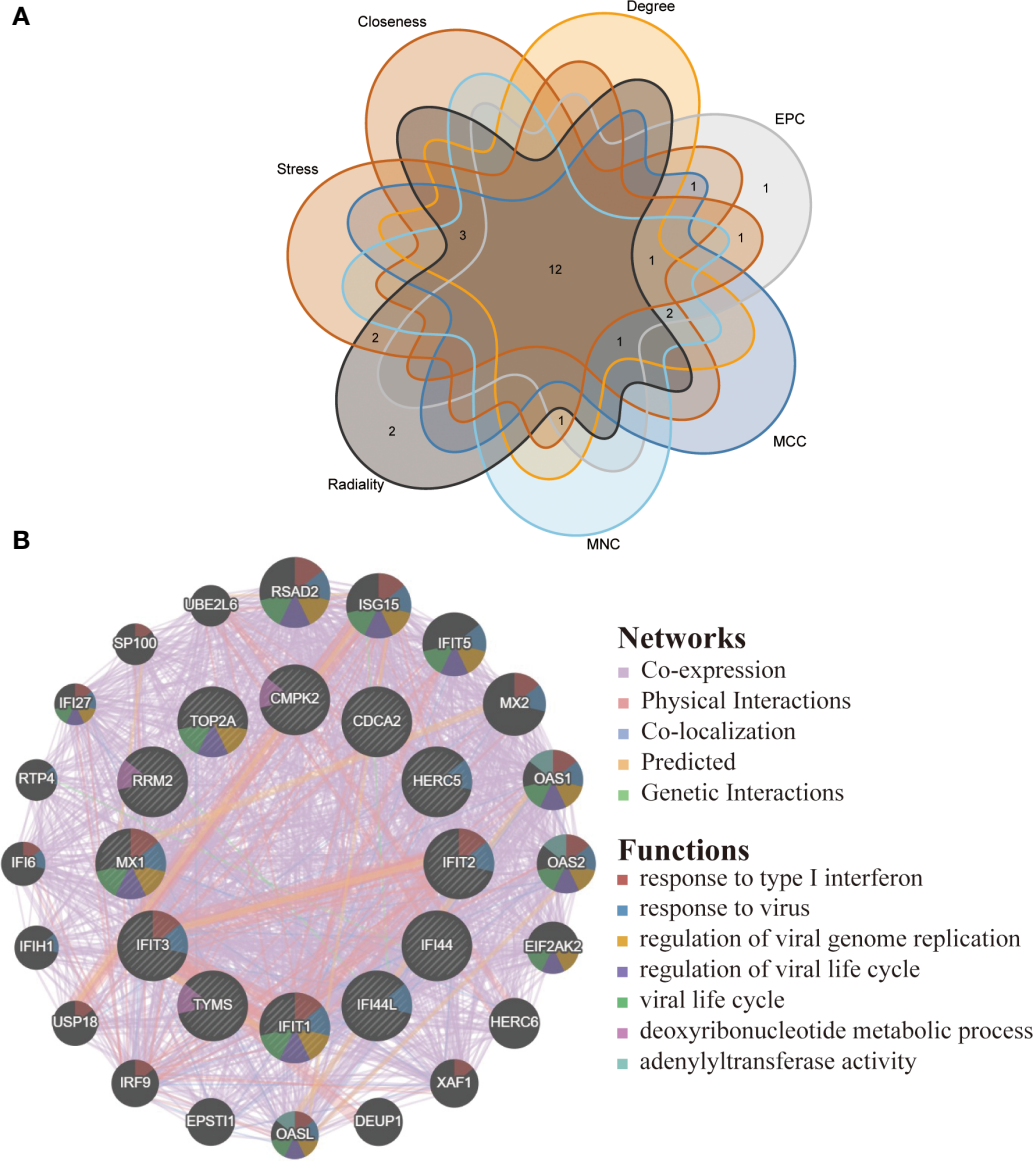
Venn diagram and co-expression network of hub genes. **(A)** The Venn diagram showed 12 overlapping hub genes screened by 7 algorithms. **(B)** Hub genes and their co-expression genes were analyzed *via* GeneMANIA.

By applying the seven algorithms of plug-in cytoHubba, we screened the top 20 hub genes. Through the intersection of Venn diagrams, 12 common hub genes were finally identified, including CMPK2, TYMS, RRM2, HERC5, IFI44L, IFI44, IFIT2, IFIT1, IFIT3, MX1, CDCA2 and TOP2A (**Figure 6A**). According to GeneMANIA database, we constructed a complex gene interaction network to decipher the biological functions of these hub genes, with the co-expression of 60.37%, physical interactions of 33.91%, co-localization of 3.46%, predicted of 2.15% and pathway of 0.10% (**Figure 6B**). Twenty genes associated with the 12 hub genes were identified, and the results showed that they were mainly linked to response to type I interferon, response to virus, regulation of viral genome replication, regulation of viral life cycle, viral life cycle, deoxyribonucleotide metabolic process and adenylyltransferase activity.

### TF-gene interactions and TF-miRNA co-regulatory network

**Figure 7:**
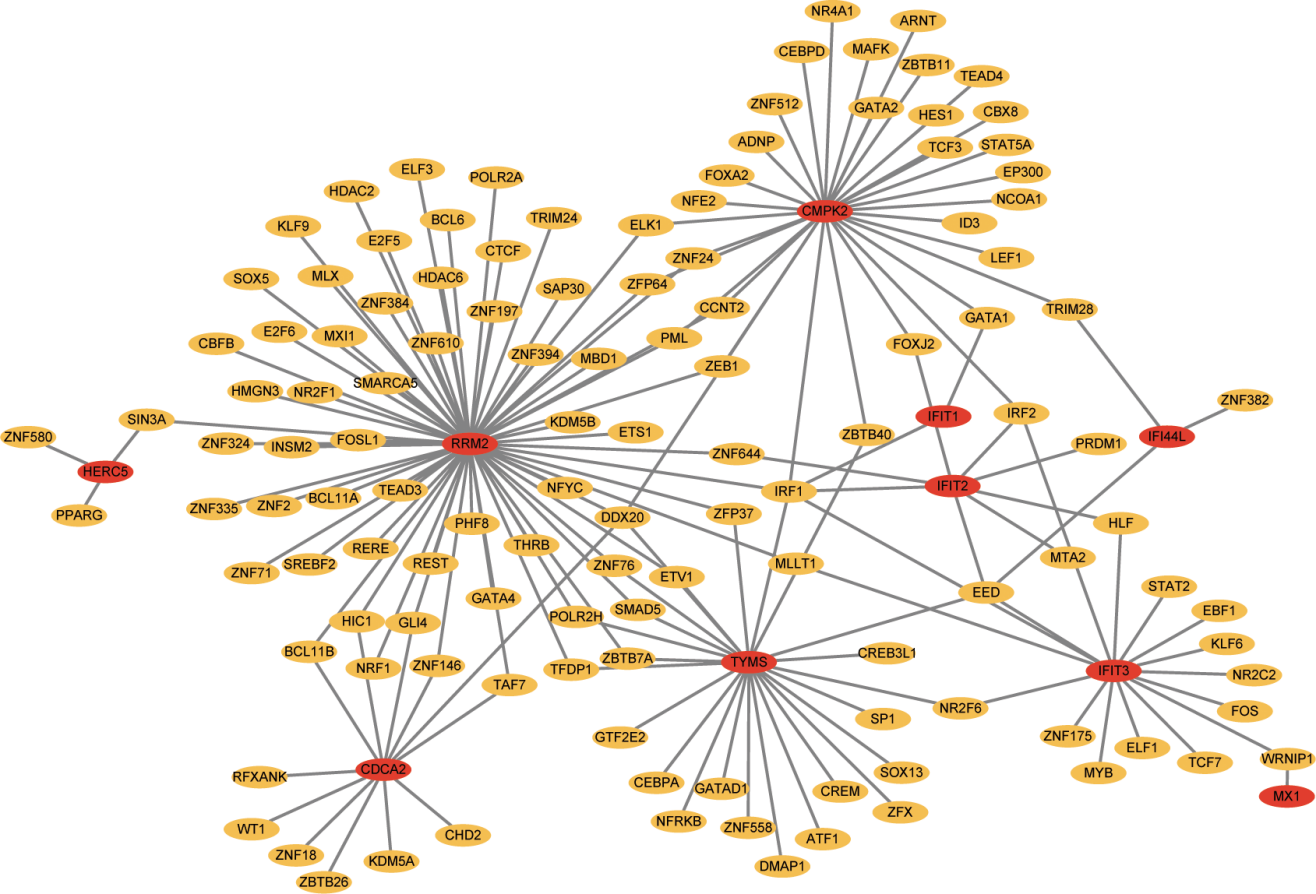
Network for TF-gene interaction with hub genes. The highlighted blue color node represents the hub genes and other nodes represent TF-genes.

**Figure 8:**
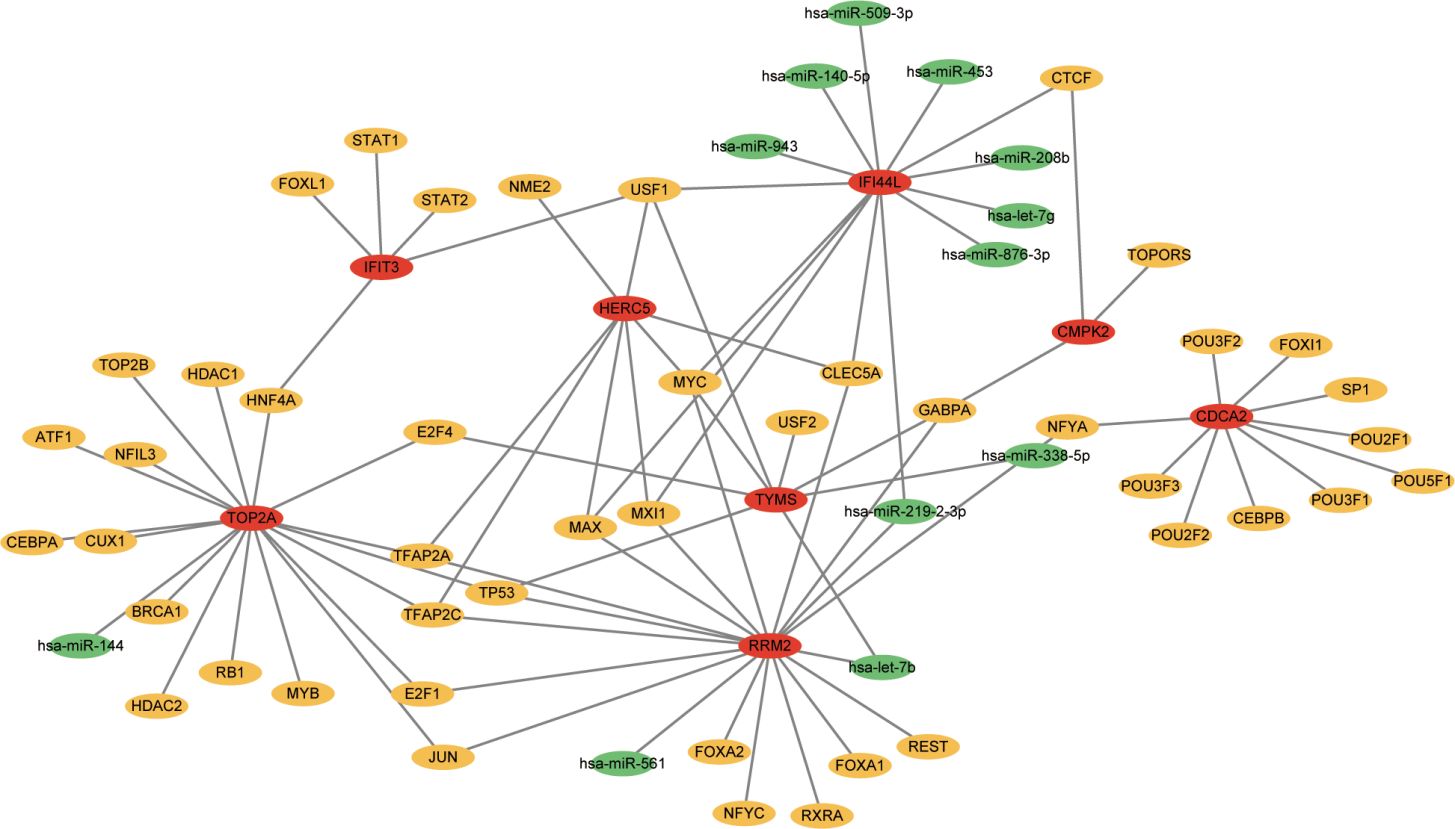
The network presents the TF-miRNA coregulatory network. The network consists of 65 nodes and 85 edges including 45 TF-genes, 12 miRNA and 8 hub genes. The nodes in red color are the hub genes, a yellow node represents TF-genes and other nodes indicate miRNAs.

TFs which can interact with the 12 hub genes were predicted by Networkanalyst, and the TF-gene regulatory network was plotted and visualized by Cytoscape (**Figure 7**). The network contains 124 TFs, 134 nodes and 165 edges. These TFs regulate more than one hub gene in the network, which demonstrated the high interaction of TFs with hub genes. Subsequently, TF-miRNA co-regulatory network was constructed using NetworkAnalyst, which predicted the interaction of miRNA and TF with hub genes (**Figure 8**). This interaction may be responsible for the regulation of hub gene expression. The network included 65 nodes and 85 edges and 12 miRNAs and 45 TF genes interacted with hub genes.

### ROC curves of hub genes

**Figure 9:**
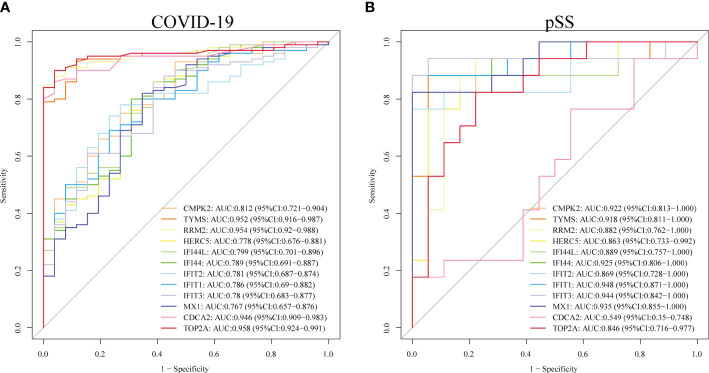
Validation of diagnostic shared biomarkers. **(A)** The ROC curve of the diagnostic efficacy verification in GSE157103. **(B)** The ROC curve of the diagnostic efficacy verification in GSE40611.

We assessed the diagnostic efficacy of the 12 hub genes by plotting ROC curves. In the COVID-19 dataset, TYMS(AUC:0.952), RRM2(AUC:0.954), CDCA2(AUC:0.946) and TOP2A(AUC:0.958) exhibited good diagnostic efficiency for differentiating the patients with SARS-CoV-2 from healthy controls (**Figure 9A**). In the pSS dataset, CMPK2 (AUC:0.922), TYMS (AUC:0.918), IFI44 (AUC:0.925), IFIT1 (AUC:0.948), IFIT3(AUC:0.944) and MX1 (AUC:0.935) exhibited preferable diagnostic performance for differentiating pSS patients from healthy controls (**Figure 9B**).

## Discussion

Evidence has indicated that the prevalence of COVID-19 was significantly higher in patients with pSS compared to the general population. A variety of viruses (EBV, CMV and HCV) have been found to be closely associated with the development of pSS, but there are still fewer studies on COVID-19 and pSS. Hence, we attempted to explore the shared molecular biological function and pathways between COVID-19 and pSS, and to determine the interrelationship between COVID-19 and pSS.

In this study, 40 shared DEGs of COVID-19 and pSS have been identified. After constructing the PPI network of common DEGs, we identified 12 hub genes (CMPK2, TYMS, RRM2, HERC5, IFI44L, IFI44, IFIT2, IFIT1, IFIT3, MX1, CDCA2 and TOP2A).

Seven genes have been reported to be related to the pathological mechanism of COVID-19 and pSS. CMPK2 (Cytidine/uridine monophosphate kinase 2) is a thymidylate kinase, known to be associated with mitochondrial DNA (mtDNA) synthesis, and may attenuate the severity of acute respiratory distress syndrome (ARDS), a common complication of severe COVID-19, by rate-limiting for mtDNA synthesis (34, 35). In pSS samples, CMPK2 was reported to be upregulated and was linked to the extent of immune cell infiltration, mitochondrial respiratory chain complexes, and mitochondrial metabolic pathways (36). HERC5 (HECT and RLD domain containing E3 ubiquitin protein ligase 5) is an antiviral immune protein which is induced by interferon. It can inhibit replication of hepatitis C (HCV), influenza A (IAV), human immunodeficiency virus (HIV), SARS-CoV-2 and other viruses by mediating ISGylation of protein targets induced by type I interferon (37–39). IFI44L (Interferon(IFN) Induced Protein 44 Like) is a type-1 I interferon-stimulated gene, induced by several different viruses. IFI44L expression was significantly higher in pSS patients than in controls and was markedly increased after IFN-α or IFN-β stimulation (40, 41). In addition, this gene was also significantly upregulated in SARS-CoV-2 infected cardiac tissues (42). As a feedback regulator of IFN responses, IFI44L can facilitate virus replication *via* modulating innate immune responses induced after virus infections (43). IFIT (Interferon-induced protein with tetratricopeptide repeats) genes are interferon-stimulated genes and consist of four genes, IFIT1, IFIT2, IFIT3 and IFIT5. The expression of IFIT genes is low in multiple cell types, while viral infection can increase their expression. In pathological conditions, they inhibit viral replication by binding and modulating the function of cellular and viral proteins (44, 45). IFIT1, IFIT2 and IFIT3 have been shown to be upregulated in cells infected with SARS-CoV-2, indicating activation of the interferon innate response, which could be regarded as potential drug targets for the treatment of COVID-19 (46–48). Moreover, they may upregulate the expression of CXCL10 which induces lymphocyte chemotaxis and may inhibit the replication of viruses. These molecules may play a critical role in the innate immune response in response to viruses (49). MX1 (Myxovirus resistance 1) encodes a guanosine triphosphate (GTP) metabolizing protein involved in the cellular antiviral response. The encoded protein is induced by type I and type II interferons and antagonizes the replication process of multiple different RNA and DNA viruses. Through binding to viral nucleoproteins, MX1 can interfere with the transcription of influenza viruses (50–52). Several studies have revealed that MX1 is overexpressed in COVID-19 group compared to control group, due to the activation of MX1 responding to new viruses for which the body has no immune defense (53–55). In addition, the baseline level of MX1 help to identify SARS-CoV-2-positive patients and help to differentiate patients who are inclined to different outcomes (56). Similarly, the expression of MX1 was significantly upregulated in the pSS (57). For the remaining five hub genes (TYMS, CDCA2, TOP2A, RRM2 and IFI44), there are no studies reporting their role in COVID-19 or pSS, which emphasizes its importance in future research.

In this study, GO enrichment analysis indicated that the type I interferon signaling pathway is common pathogenesis of COVID-19 and pSS. Furthermore, based on published publications, we hypothesized that type I interferon might be a shared mechanism of COVID-19 and pSS.

Interferon (IFN) is a class of cytokines with antiviral effects that directly induce anti-pathogenic immune responses by controlling the inflammatory response and coordinating the immune response, thereby resisting invasion and infection by foreign pathogens (58). Interferons induced by viral infections can be produced through different signaling pathways, eventually leading to the transcription and expression of hundreds of IFN-stimulated genes (ISGs), which further exert antiviral effects (59). Interferons are classified into three major classes: type I, II, and III interferons. Type I interferons (IFN-α, IFN-β, IFN-ϵ, IFN-κ, IFN-ω) are secreted by virus-infected cells, type II interferons (IFN-γ) are secreted by activated T cells, and type III IFN (IFN-λ) binds to type III IFN receptors (IFNLR) and is preferentially expressed on epithelial cells and certain bone marrow cells (60). Type I interferon is the main type of interferon that can exert antiviral effects.

Although IFN has anti-multiviral effects, it does not kill viruses directly, but rather inhibits the replication process by producing antiviral proteins (61). Studies have demonstrated that IFN can induce the expression of antiviral proteins upon viral infection (62). IFN-α can significantly enhance cellular susceptibility to microorganisms by upregulating Toll-like receptors (TLRs) expression or the expression of transduction molecules and kinases involved in TLR signaling (63). Moreover, IFN-α strongly increases the differentiation of T cells and enhances cellular immunity (64). In addition to its effect on T cells, IFN can also promote the proliferation of B cells and enhance humoral immune responses (65). In summary, there are two main antiviral mechanisms of IFN: one is acting on viruses, such as interfering with viral replication, and the other is acting on cells to strengthen the immune function of the body.

There is substantial evidence that type I interferon plays an important role in the pathogenesis and progression of pSS due to immune dysregulation (66). For example, it can influence the immune response to pSS, participating in the activation of antiviral responses and controlling immune responses through interactions with the corresponding receptors (67). An important role of type I interferon is to induce immune activation, which affects the production and regulation of pro-inflammatory cytokines and other mediators (68). Monocytes are stimulated by type I interferon to differentiate into dendritic cells and stimulate immature dendritic cells to express chemokines and costimulatory molecules that facilitate their homing to secondary lymphoid organs, thereby activating adaptive immunity (69). In addition, macrophages are stimulated by interferon to enhance phagocytosis. BAFF (B cell activating factor) is known to be involved in the pathogenesis of pSS, as it is upregulated in monocytes in response to type I and type II IFN and promotes B-cell survival (70). In addition to monocytes, macrophages, dendritic cells and salivary gland epithelial cells also express BAFF in response to IFN stimulation (71, 72). Transgenic mice that overproduce BAFF exhibit increased B cell proliferation, increased germinal center responses, autoantibody production, and increased numbers of immune complexes (73). This suggests that IFN (especially IFN type I) promotes the development of pSS by inducing innate immunity, activating adaptive immunity, and regulating inflammatory cytokines and antibody levels.

Although previous studies have explored the pivotal genes associated with COVID-19 and pSS, respectively. However, few studies have explored the common molecular mechanisms between the two through bioinformatic approaches. In this study, we explored and identified common DEGs, hub genes and TFs of COVID-19 and pSS for the first time, which helped to further elucidate the pathogenesis of both. However, our study also has some limitations. First, this study requires external validation to verify our findings; second, the function of hub genes needs to be further validated in an *in vitro* model, which will be the focus of our future work.

In conclusion, we identified common DEGs for COVID-19 and pSS and performed enrichment and PPI network analysis. We found that COVID-19 and pSS share pathogenic mechanism that may be mediated by specific hub genes. This study provides a potential direction for further investigation of the molecular mechanisms of COVID-19 and pSS.

## Data Availability Statement

The datasets presented in this study can be found in online repositories. The names of the repository/repositories and accession number(s) can be found in the article/supplementary material.

## Author Contributions

HL performed data analyses and wrote the manuscript draft. XZ revised the manuscript. All authors read and approved the final manuscript. All authors contributed to the article and approved the submitted version.

## Funding

This research was supported financially by the Funding of Wuhan Health Planning Commission Program (WX15B21, to XZ).

## Acknowledgements

We thank the authors of the GSE157103 and GSE40611 datasets for their contribution.

## Conflict of Interest

The authors declare that the research was conducted in the absence of any commercial or financial relationships that could be construed as a potential conflict of interest.

## Publisher’s Note

All claims expressed in this article are solely those of the authors and do not necessarily represent those of their affiliated organizations, or those of the publisher, the editors and the reviewers. Any product that may be evaluated in this article, or claim that may be made by its manufacturer, is not guaranteed or endorsed by the publisher.

## References

[1] Mavragani CP. Mechanisms and new strategies for primary sjögren's syndrome. Annu Rev Med (2017) 68:331–43. doi: 10.1146/annurev-med-043015-123313 28099084

[2] Li N Li L Wu M Li Y Yang J Wu Y. Integrated bioinformatics and validation reveal potential biomarkers associated with progression of primary sjögren's syndrome. Front Immunol (2021) 12:697157. doi: 10.3389/fimmu.2021.697157 34367157 PMC8343000

[3] Qin B Wang J Yang Z Yang M Ma N Huang F. Epidemiology of primary sjögren's syndrome: A systematic review and meta-analysis. Ann Rheum Dis (2015) 74(11):1983–9. doi: 10.1136/annrheumdis-2014-205375 24938285

[4] Fox RI. Sjögren's syndrome. Lancet (2005) 366(9482):321–31. doi: 10.1016/s0140-6736(05)66990-5 16039337

[5] Kapsogeorgou EK Voulgarelis M Tzioufas AG. Predictive markers of lymphomagenesis in sjögren's syndrome: From clinical data to molecular stratification. J Autoimmun (2019) 104:102316. doi: 10.1016/j.jaut.2019.102316 31431317

[6] Draborg A Izarzugaza JM Houen G. How compelling are the data for Epstein-Barr virus being a trigger for systemic lupus and other autoimmune diseases? Curr Opin Rheumatol (2016) 28(4):398–404. doi: 10.1097/bor.0000000000000289 26986247

[7] Saito I Servenius B Compton T Fox RI. Detection of Epstein-barr virus DNA by polymerase chain reaction in blood and tissue biopsies from patients with sjogren's syndrome. J Exp Med (1989) 169(6):21941–8. doi: 10.1084/jem.169.6.2191 PMC2189340 2543732

[8] Liu X Sadaoka T Krogmann T Cohen JI. Epstein-Barr Virus (Ebv) tegument protein Bglf2 suppresses type I interferon signaling to promote ebv reactivation. J Virol (2020) 94(11):e00258–20. doi: 10.1128/jvi.00258-20 32213613 PMC7269453

[9] Shimizu T Nakamura H Takatani A Umeda M Horai Y Kurushima S. Activation of toll-like receptor 7 signaling in labial salivary glands of primary sjögren's syndrome patients. Clin Exp Immunol (2019) 196(1):39–51. doi: 10.1111/cei.13242 30446998 PMC6422652

[10] Navone R Lunardi C Gerli R Tinazzi E Peterlana D Bason C. Identification of tear lipocalin as a novel autoantigen target in sjögren's syndrome. J Autoimmun (2005) 25(3):229–34. doi: 10.1016/j.jaut.2005.09.021 16249071

[11] Croia C Astorri E Murray-Brown W Willis A Brokstad KA Sutcliffe N. Implication of Epstein-Barr virus infection in disease-specific autoreactive b cell activation in ectopic lymphoid structures of sjögren's syndrome. Arthritis Rheumatol (Hoboken NJ) (2014) 66(9):2545–57. doi: 10.1002/art.38726 24891330

[12] Kivity S Arango MT Ehrenfeld M Tehori O Shoenfeld Y Anaya JM. Infection and autoimmunity in sjogren's syndrome: A clinical study and comprehensive review. J Autoimmun (2014) 51:17–22. doi: 10.1016/j.jaut.2014.02.008 24637076

[13] Haddad J Deny P Munz-Gotheil C Ambrosini JC Trinchet JC Pateron D. Lymphocytic sialadenitis of sjögren's syndrome associated with chronic hepatitis c virus liver disease. Lancet (1992) 339(8789):321–3. doi: 10.1016/0140-6736(92)91645-o PMC7134660 1346409

[14] Huang C Wang Y Li X Ren L Zhao J Hu Y. Clinical features of patients infected with 2019 novel coronavirus in wuhan, China. Lancet (2020) 395(10223):497–506. doi: 10.1016/s0140-6736(20)30183-5 31986264 PMC7159299

[15] Ferri C Giuggioli D Raimondo V L'Andolina M Tavoni A Cecchetti R. Covid-19 and rheumatic autoimmune systemic diseases: Report of a Large Italian patients series. Clin Rheumatol (2020) 39(11):3195–204. doi: 10.1007/s10067-020-05334-7 PMC7450255 32852623

[16] Chowdhury F Grigoriadou S Bombardieri M. Severity of covid-19 infection in primary sjögren's syndrome and the emerging evidence of covid-19-Induced xerostomia. Clin Exp Rheumatol (2021) 39 Suppl 133(6):215–22. doi: 10.55563/clinexprheumatol/k7x3ta 34919045

[17] Akiyama S Hamdeh S Micic D Sakuraba A. Prevalence and clinical outcomes of covid-19 in patients with autoimmune diseases: A systematic review and meta-analysis. Ann Rheum Dis (2020) 80(3):384–91. doi: 10.1136/annrheumdis-2020-218946 33051220

[18] Saadoun D Vieira M Vautier M Baraliakos X Andreica I da Silva JAP. Sars-Cov-2 outbreak in immune-mediated inflammatory diseases: The Euro-covimid multicentre cross-sectional study. Lancet Rheumatol (2021) 3(7):e481–e8. doi: 10.1016/s2665-9913(21)00112-0 PMC8081401 33942031

[19] Barrett T Troup DB Wilhite SE Ledoux P Evangelista C Kim IF. Ncbi geo: Archive for functional genomics data sets–10 years on. Nucleic Acids Res (2011) 39(Database issue):D1005–10. doi: 10.1093/nar/gkq1184 PMC3013736 21097893

[20] Overmyer KA Shishkova E Miller IJ Balnis J Bernstein MN Peters-Clarke TM. Large-Scale multi-omic analysis of covid-19 severity. Cell Syst (2021) 12(1):23–40.e7. doi: 10.1016/j.cels.2020.10.003 33096026 PMC7543711

[21] Horvath S Nazmul-Hossain AN Pollard RP Kroese FG Vissink A Kallenberg CG. Systems analysis of primary sjögren's syndrome pathogenesis in salivary glands identifies shared pathways in human and a mouse model. Arthritis Res Ther (2012) 14(6):R238. doi: 10.1186/ar4081 23116360 PMC3674589

[22] Barrett T Wilhite SE Ledoux P Evangelista C Kim IF Tomashevsky M. Ncbi geo: Archive for functional genomics data sets–update. Nucleic Acids Res (2013) 41(Database issue):D991–5. doi: 10.1093/nar/gks1193 PMC3531084 23193258

[23] Xia J Benner MJ Hancock RE. Networkanalyst–integrative approaches for protein-protein interaction network analysis and visual exploration. Nucleic Acids Res (2014) 42(Web Server issue):W167–74. doi: 10.1093/nar/gku443 PMC4086107 24861621

[24] Chen H Boutros PC. Venndiagram: A package for the generation of highly-customizable Venn and Euler diagrams in r. BMC Bioinf (2011) 12:35. doi: 10.1186/1471-2105-12-35 PMC3041657 21269502

[25] Bu D Luo H Huo P Wang Z Zhang S He Z. Kobas-I: Intelligent prioritization and exploratory visualization of biological functions for gene enrichment analysis. Nucleic Acids Res (2021) 49(W1):W317–25. doi: 10.1093/nar/gkab447 PMC8265193 34086934

[26] Szklarczyk D Gable AL Lyon D Junge A Wyder S Huerta-Cepas J. String V11: Protein-protein association networks with increased coverage, supporting functional discovery in genome-wide experimental datasets. Nucleic Acids Res (2019) 47(D1):D607–13. doi: 10.1093/nar/gky1131 PMC6323986 30476243

[27] Shannon P Markiel A Ozier O Baliga NS Wang JT Ramage D. Cytoscape: A software environment for integrated models of biomolecular interaction networks. Genome Res (2003) 13(11):2498–504. doi: 10.1101/gr.1239303 PMC403769 14597658

[28] Bader GD Hogue CW. An automated method for finding molecular complexes in Large protein interaction networks. BMC Bioinf (2003) 4:2. doi: 10.1186/1471-2105-4-2 PMC149346 12525261

[29] Chin CH Chen SH Wu HH Ho CW Ko MT Lin CY. Cytohubba: Identifying hub objects and Sub-networks from complex interactome. BMC Syst Biol (2014) 8 Suppl 4(Suppl 4):S11. doi: 10.1186/1752-0509-8-s4-s11 25521941 PMC4290687

[30] Franz M Rodriguez H Lopes C Zuberi K Montojo J Bader GD. Genemania update 2018. Nucleic Acids Res (2018) 46(W1):W60–4. doi: 10.1093/nar/gky311 PMC6030815 29912392

[31] Davis CA Hitz BC Sloan CA Chan ET Davidson JM Gabdank I. The encyclopedia of DNA elements (Encode): Data portal update. Nucleic Acids Res (2018) 46(D1):D794–801. doi: 10.1093/nar/gkx1081 29126249 PMC5753278

[32] Liu ZP Wu C Miao H Wu H. Regnetwork: An integrated database of transcriptional and post-transcriptional regulatory networks in human and mouse. Database (Oxford) (2015) 2015:bav095. doi: 10.1093/database/bav095 26424082 PMC4589691

[33] Robin X Turck N Hainard A Tiberti N Lisacek F Sanchez JC. Proc: An open-source package for r and s+ to analyze and compare roc curves. BMC Bioinf (2011) 12:77. doi: 10.1186/1471-2105-12-77 PMC3068975 21414208

[34] Feng C Tang Y Liu X Zhou Z. Cmpk2 of triploid crucian carp is involved in immune defense against bacterial infection. Dev Comp Immunol (2021) 116:103924. doi: 10.1016/j.dci.2020.103924 33186560

[35] Xian H Liu Y Rundberg Nilsson A Gatchalian R Crother TR Tourtellotte WG. Metformin inhibition of mitochondrial atp and DNA synthesis abrogates Nlrp3 inflammasome activation and pulmonary inflammation. Immunity (2021) 54(7):1463–77.e11. doi: 10.1016/j.immuni.2021.05.004 34115964 PMC8189765

[36] Li N Li Y Hu J Wu Y Yang J Fan H. A link between mitochondrial dysfunction and the immune microenvironment of salivary glands in primary sjogren's syndrome. Front Immunol (2022) 13:845209. doi: 10.3389/fimmu.2022.845209 35359935 PMC8964148

[37] Xue F Higgs BW Huang J Morehouse C Zhu W Yao X. Herc5 is a prognostic biomarker for post-liver transplant recurrent human hepatocellular carcinoma. J Transl Med (2015) 13:379. doi: 10.1186/s12967-015-0743-2 26653219 PMC4676172

[38] Paparisto E Woods MW Coleman MD Moghadasi SA Kochar DS Tom SK. Evolution-guided structural and functional analyses of the herc family reveal an ancient marine origin and determinants of antiviral activity. J Virol (2018) 92(13):e00528–18. doi: 10.1128/jvi.00528-18 29669830 PMC6002735

[39] Mathieu NA Paparisto E Barr SD Spratt DE. Herc5 and the isgylation pathway: Critical modulators of the antiviral immune response. Viruses (2021) 13(6):1102. doi: 10.3390/v13061102 34207696 PMC8228270

[40] Zhao M Zhou Y Zhu B Wan M Jiang T Tan Q. Ifi44l promoter methylation as a blood biomarker for systemic lupus erythematosus. Ann Rheum Dis (2016) 75(11):1998–2006. doi: 10.1136/annrheumdis-2015-208410 26787370 PMC4955646

[41] Jara D Carvajal P Castro I Barrera MJ Aguilera S González S. Type I interferon dependent hsa-Mir-145-5p downregulation modulates Muc1 and Tlr4 overexpression in salivary glands from sjögren's syndrome patients. Front Immunol (2021) 12:685837. doi: 10.3389/fimmu.2021.685837 34149728 PMC8208490

[42] Bräuninger H Stoffers B Fitzek ADE Meißner K Aleshcheva G Schweizer M. Cardiac sars-Cov-2 infection is associated with pro-inflammatory transcriptomic alterations within the heart. Cardiovasc Res (2022) 118(2):542–55. doi: 10.1093/cvr/cvab322 PMC8803085 34647998

[43] DeDiego ML Martinez-Sobrido L Topham DJ. Novel functions of Ifi44l as a feedback regulator of host antiviral responses. J Virol (2019) 93(21):e01159–19. doi: 10.1128/jvi.01159-19 PMC6803278 31434731

[44] Pidugu VK Pidugu HB Wu MM Liu CJ Lee TC. Emerging functions of human ifit proteins in cancer. Front Mol Biosci (2019) 6:148. doi: 10.3389/fmolb.2019.00148 31921891 PMC6930875

[45] Fensterl V Sen GC. Interferon-induced ifit proteins: Their role in viral pathogenesis. J Virol (2015) 89(5):2462–8. doi: 10.1128/jvi.02744-14 PMC4325746 25428874

[46] Vishnubalaji R Shaath H Alajez NM. Protein coding and long noncoding rna (Lncrna) transcriptional landscape in sars-Cov-2 infected bronchial epithelial cells highlight a role for interferon and inflammatory response. Genes (2020) 11(7):760. doi: 10.3390/genes11070760 PMC7397219 32646047

[47] Caldarale F Giacomelli M Garrafa E Tamassia N Morreale A Poli P. Plasmacytoid dendritic cells depletion and elevation of ifn-Γ dependent chemokines Cxcl9 and Cxcl10 in children with multisystem inflammatory syndrome. Front Immunol (2021) 12:654587. doi: 10.3389/fimmu.2021.654587 33841438 PMC8033149

[48] Prasad K Khatoon F Rashid S Ali N AlAsmari AF Ahmed MZ. Targeting hub genes and pathways of innate immune response in covid-19: A network biology perspective. Int J Biol Macromol (2020) 163:1–8. doi: 10.1016/j.ijbiomac.2020.06.228 32599245 PMC7319641

[49] Imaizumi T Hashimoto S Sato R Umetsu H Aizawa T Watanabe S. Ifit proteins are involved in Cxcl10 expression in human glomerular endothelial cells treated with a toll-like receptor 3 agonist. Kidney Blood Press Res (2021) 46(1):74–83. doi: 10.1159/000511915 33326977

[50] Aljohani AI Joseph C Kurozumi S Mohammed OJ Miligy IM Green AR. Myxovirus resistance 1 (Mx1) is an independent predictor of poor outcome in invasive breast cancer. Breast Cancer Res Treat (2020) 181(3):541–51. doi: 10.1007/s10549-020-05646-x PMC7220876 32350677

[51] Verhelst J Hulpiau P Saelens X. Mx proteins: Antiviral gatekeepers that restrain the uninvited. Microbiol Mol Biol Rev (2013) 77(4):551–66. doi: 10.1128/mmbr.00024-13 PMC3973384 24296571

[52] Pillai PS Molony RD Martinod K Dong H Pang IK Tal MC. Mx1 reveals innate pathways to antiviral resistance and lethal influenza disease. Science (2016) 352(6284):463–6. doi: 10.1126/science.aaf3926 PMC5465864 27102485

[53] Haller O Staeheli P Schwemmle M Kochs G. Mx gtpases: Dynamin-like antiviral machines of innate immunity. Trends Microbiol (2015) 23(3):154–63. doi: 10.1016/j.tim.2014.12.003 25572883

[54] Bizzotto J Sanchis P Abbate M Lage-Vickers S Lavignolle R Toro A. Sars-Cov-2 infection boosts Mx1 antiviral effector in covid-19 patients. iScience (2020) 23(10):101585. doi: 10.1016/j.isci.2020.101585 32989429 PMC7510433

[55] Verhelst J Parthoens E Schepens B Fiers W Saelens X. Interferon-inducible protein Mx1 inhibits influenza virus by interfering with functional viral ribonucleoprotein complex assembly. J Virol (2012) 86(24):13445–55. doi: 10.1128/jvi.01682-12 PMC3503048 23015724

[56] Maras JS Sharma S Bhat A Rooge S Aggrawal R Gupta E. Multi-omics analysis of respiratory specimen characterizes baseline molecular determinants associated with sars-Cov-2 outcome. iScience (2021) 24(8):102823. doi: 10.1016/j.isci.2021.102823 34308298 PMC8268673

[57] Papinska J Bagavant H Gmyrek GB Deshmukh US. Pulmonary involvement in a mouse model of sjögren's syndrome induced by sting activation. Int J Mol Sci (2020) 21(12):4512. doi: 10.3390/ijms21124512 PMC7349948 32630417

[58] Wang W Xu L Su J Peppelenbosch MP Pan Q. Transcriptional regulation of antiviral interferon-stimulated genes. Trends Microbiol (2017) 25(7):573–84. doi: 10.1016/j.tim.2017.01.001 PMC7127685 28139375

[59] Park A Iwasaki A. Type I and type iii interferons - induction, signaling, evasion, and application to combat covid-19. Cell Host Microbe (2020) 27(6):870–8. doi: 10.1016/j.chom.2020.05.008 PMC7255347 32464097

[60] Kotenko SV Rivera A Parker D Durbin JE. Type iii ifns: Beyond antiviral protection. Semin Immunol (2019) 43:101303. doi: 10.1016/j.smim.2019.101303 31771761 PMC7141597

[61] Zhang R Tang J. Evasion of I interferon-mediated innate immunity by pseudorabies virus. Front Microbiol (2021) 12:801257. doi: 10.3389/fmicb.2021.801257 34970252 PMC8712723

[62] Liu SY Sanchez DJ Cheng G. New developments in the induction and antiviral effectors of type I interferon. Curr Opin Immunol (2011) 23(1):57–64. doi: 10.1016/j.coi.2010.11.003 21123041 PMC3822007

[63] Mitchell D Chintala S Dey M. Plasmacytoid dendritic cell in immunity and cancer. J Neuroimmunol (2018) 322:63–73. doi: 10.1016/j.jneuroim.2018.06.012 30049538

[64] Chenna Narendra S Chalise JP Biggs S Kalinke U Magnusson M. Regulatory T-cells mediate ifn-A-Induced resistance against antigen-induced arthritis. Front Immunol (2018) 9:285. doi: 10.3389/fimmu.2018.00285 29515584 PMC5826073

[65] Graalmann T Borst K Manchanda H Vaas L Bruhn M Graalmann L. B cell depletion impairs vaccination-induced Cd8(+) T cell responses in a type I interferon-dependent manner. Ann Rheum Dis (2021) 80(12):1537–44. doi: 10.1136/annrheumdis-2021-220435 PMC8600602 34226189

[66] Del Papa N Minniti A Lorini M Carbonelli V Maglione W Pignataro F. The role of interferons in the pathogenesis of sjögren's syndrome and future therapeutic perspectives. Biomolecules (2021) 11(2):251. doi: 10.3390/biom11020251 33572487 PMC7916411

[67] Lee AJ Ashkar AA. The dual nature of type I and type ii interferons. Front Immunol (2018) 9:2061. doi: 10.3389/fimmu.2018.02061 30254639 PMC6141705

[68] Bodewes ILA Versnel MA. Interferon activation in primary sjögren's syndrome: Recent insights and future perspective as novel treatment target. Expert Rev Clin Immunol (2018) 14(10):817–29. doi: 10.1080/1744666x.2018.1519396 30173581

[69] Luo S Wu R Li Q Zhang G. Epigenetic regulation of Ifi44l expression in monocytes affects the functions of monocyte-derived dendritic cells in systemic lupus erythematosus. J Immunol Res (2022) 2022:4053038. doi: 10.1155/2022/4053038 35592687 PMC9113863

[70] Nocturne G Mariette X. B cells in the pathogenesis of primary sjögren syndrome. Nat Rev Rheumatol (2018) 14(3):133–45. doi: 10.1038/nrrheum.2018.1 29416129

[71] Bron AJ de Paiva CS Chauhan SK Bonini S Gabison EE Jain S. Tfos dews ii pathophysiology report. Ocular Surface (2017) 15(3):438–510. doi: 10.1016/j.jtos.2017.05.011 28736340

[72] Kiefer K Oropallo MA Cancro MP Marshak-Rothstein A. Role of type I interferons in the activation of autoreactive b cells. Immunol Cell Biol (2012) 90(5):498–504. doi: 10.1038/icb.2012.10 22430248 PMC3701256

[73] Thompson N Isenberg DA Jury EC Ciurtin C. Exploring baff: Its expression, receptors and contribution to the immunopathogenesis of sjögren's syndrome. Rheumatol (Oxford) (2016) 55(9):1548–55. doi: 10.1093/rheumatology/kev420 26790457

